# The Impact of Economy on Carbon Emissions: An Empirical Study Based on the Synergistic Effect of Gender Factors

**DOI:** 10.3390/ijerph16193723

**Published:** 2019-10-02

**Authors:** Shiran Li, Hongbing Deng, Kangkang Zhang

**Affiliations:** 1 School of Economics and Management, China University of Geosciences, Wuhan 430074, China; l2018sr@163.com (S.L.); denghongbing_2005@126.com (H.D.); 2 Hubei Institutes of Soft Science on Regional Innovation Capacity Monitoring and Analysis, Wuhan 430074, China

**Keywords:** carbon emissions, economy, gender factors, spatial econometrics, Bayesian model, synergy effects

## Abstract

The study of carbon emissions is of great significance for environmental change and economic development. Gender factors is an important perspective to examine the path of carbon emissions. Based on the panel data of 30 provinces in China from 2005 to 2016, this paper selects the optimal spatial measurement model structure by using the Bayesian posterior probability model structure selection method, and studies the impact of economy on carbon emissions and the influence mechanism of gender-based “synergy effect” on carbon emissions from the National level and regional levels. The research shows that the increase of economic promotes the increase of carbon emission in this region, but it has a restraining effect on the carbon emission in the surrounding areas. Moreover, gender factors have a significant positive effect on the region at the National level and the Eastern and Northeastern regions, but not significantly in other ones, and have a significant negative impact on carbon emissions in surrounding areas. Overall, the influence intensity of economy on carbon emission increases with the increase of gender in the National level and the Eastern and Northeastern, while the influence intensity of economy of peripheral regions on carbon emission in Central Region decreases with the increase of gender factors in peripheral regions.

## 1. Introduction

Since the industrial revolution, the industrial economy with high energy consumption and high emissions has grown rapidly and has become the main driving force for the sustained growth of the world economy. However, long-term dependence on the industrial economy to promote economic growth will inevitably consume a large amount of fossil energy, resulting in a continuous increase in total carbon emissions [1]. Since the reform and opening up, the two-wheel drive of industrialization and urbanization has promoted the rapid growth of China’s economy, from 1979 to 2016, the average annual growth rate of GDP was as high as 15%. Statistics show that in 2000, China’s per capita carbon emissions were 2.697 tons, while in 2011, per capita carbon emissions increased by nearly three times, reaching 7.242 tons. China’s total energy consumption in 2016 was 4.36 billion tons of standard coal, and its carbon dioxide emissions reached 10.21 billion tons in an unprecedented way, making it the world’s largest carbon dioxide emitter. The continuous accumulation of carbon emissions creates a range of environmental problems, such as global warming, rising sea levels and frequent outbreaks of extreme weather. From the birth of the *United Nations Framework Convention on Climate Change* to the signing of the *Kyoto Protocol* and the convening of the *Copenhagen Climate Conference*, a series of environmental problems caused by carbon emissions have received increasing attention from all countries. Therefore, carbon emission reduction and climate change have become important issues in today’s world. However, population and climate change are closely related [2]. Factors such as population size, population age structure, gender structure, population urbanization, economic development and industrial restructuring will have an impact on climate change and carbon emission reduction. The organic combination of climate change and gender equality also raises many important research issues. Research by Cook et al. shows that in the process of dealing with climate change, gender quotas should be introduced to allow more women to participate in climate policy formulation, which not only promotes gender equality, but also improves policy effectiveness [3]. Therefore, from the perspective of the synergy between GDP and gender factors, this paper has an important practical significance to indirectly explore the effects of carbon emissions.

The existing literature has some empirical research on carbon emissions and its influencing factors, and scholars are expected to control the corresponding factors to achieve emission reduction effects. However, most scholars explore the influencing factors of carbon emissions from the perspective of direct effects, and less literature discusses the indirect effects of gender factors on carbon emissions. Scholars mainly studied the effects of energy structure, energy intensity, industrial structure, total output value and population factors on carbon emissions [4,5,6,7,8,9,10,11,12]. Japanese scholar Kaya believes that the driving force of carbon emissions in a country or region mainly includes four major factors, namely, population, per capita GDP, energy consumption per unit of GDP, carbon emissions per unit of energy consumption, and the famous Kaya formula is proposed [13]. Among them, the relationship between economic growth and carbon emissions cannot be ignored. Scholars combine macroeconomic issues such as economic growth, industrial structure and total factor productivity with carbon emissions, and incorporate carbon emission issues into the model, while extending some related concepts, such as green total factor productivity and green GDP [14,15]. Economic growth affects the total amount of carbon emissions, but in turn, the amount of carbon emissions reflects the degree of economic development, there is a mutual two-way causal relationship between the two, and actively developing a green economy can effectively reduce carbon dioxide emissions. With the rapid economic growth, the marginal carbon emissions show a downward trend, and the economic aggregates become the main driving factor for the increase of carbon emissions. At the same time, the economic growth rate contributes 42.9% to China’s per capita carbon emissions, showing an exponential growth [16,17].

As the relationship between population and resources and the environment becomes more prominent, population factors are one of the main factors affecting carbon emissions. The main way influencing the impact of demographic changes on carbon emissions is the abundant supply of labor in the production sector [18], a large number of studies have focused on the analysis of population size, economic level, technology and industrial structure using the STIRPAT model, and the results show that population size is one of the most important factors affecting carbon emissions [19,20]. Li et al. found that the population has a significant positive impact on carbon emissions, and there is a long-term, stable relationship [21]. Wang et al. also found that the population size of most provinces has a positive impact on carbon emissions [22], this is consistent with most research findings. The population ageing and gender factors in the population structure also have a significant impact on carbon emissions. The aging of the population has a negative effect on carbon dioxide emissions, and the acceleration of population aging has a negative effect on long-term carbon emissions [23]. In the study of the impact of population factors on carbon emissions, it is a very important research perspective to examine the root causes of climate change based on gender structure. Men’s “carbon footprint” is much higher than that of women, mainly due to differences in consumption and risk perception between women and men [24], and the impact of gender differences on carbon emissions is also related to the level of economic and social development [25]. Women are not only vulnerable groups in climate change, but women’s lifestyles and concepts can play a positive role in reducing emissions, and carbon emissions are sensitive to changes in gender structure, carbon emissions are significantly negatively correlated with female population [2].

Through in-depth analysis of domestic and foreign literature on carbon emissions related research, it is found that in terms of research methods, there are mainly two types of literatures: non-spatial features and spatial features. In the study of factors affecting carbon emissions without spatial characteristics, the research methods mainly include the logarithmic mean weight Divisia decomposition method (LMD) [26,27], the structural decomposition analysis method (SDA) and input and output (IO) method [28,29], the Kaya equation and the modified STIRPAT method [30,31,32], and the Environmental Kuznets Curve (EKC).

With the strengthening of inter-provincial cooperation, especially the establishment of economic circles, economic belts, population cross-regional mobility, and the addition of spatial factors, the relationship between gender and carbon emissions can be studied more effectively. In the study of carbon emission factors including spatial features, two methods are mainly considered. One method is the measure of spatial linkage characteristics, mainly the spatial correlation analysis (Moran’s I index). Fu et al., Ma et al., Cheng et al. and Wu et al. used this method to study the relationship between carbon emissions and carbon emission intensity in various provinces, and analyzed the changing relationship between carbon emissions in various provinces [9,33,34,35]. Spatial correlation analysis can calculate the relationship between different regions in a more intuitive way, and it is also an important basis for judging whether the model is added to spatial features. In addition, the LISA map provides a clear analysis of the relationship between local carbon emissions. Another method is a model that contains spatial effects in the model. The spatial effect model is mainly based on the analysis of spatial correlation. By observing the results of spatial correlation analysis to determine whether the carbon emissions have spatial correlation between regions, that is, the absolute value of the Moran’s I index is not equal to zero. In the model containing spatial effects, there are mainly two parts, and one part is the setting of the spatial weight matrix. There are many ways to set spatial weight matrix, including proximity, distance, and economic relationships. The other part is the spatial measurement model, which can be divided into four categories, namely spatial lag model (SAR or SLM), spatial error model (SEM), spatial Durbin model (SDM) and spatial Durbin error model (SDEM). Ma et al. compared the analysis results of SAR and SEM with common panel models and found that models with spatial effects can be better explained [34]. Cheng et al. used SDM model to study the influencing factors of China’s energy consumption carbon emission intensity [9]. Based on the STIRPAT model and the dynamic spatial Durbin panel data model, Niu and Liu empirically analyzed the influencing factors of China’s construction industry carbon emissions based on the 2002-2013 provincial panel data [36]. Zhao et al. measured the dynamic trend and agglomeration characteristics of carbon emission intensity in China from 2000 to 2015 by nuclear density distribution and Moran index, and analyzed the main influencing factors by using the spatial Durbin model [37].

The existing literature provides valuable references for the research of this paper. Based on the existing literature, there is still room for improvement in the impact of carbon dioxide emissions from the perspective of GDP and gender-based synergies: (1) The analysis of the influencing factors of carbon emissions in the existing literature is mainly attributed to energy structure, energy intensity, industrial structure, total output value and population factors, less interaction between gender factors and GDP is introduced, and the research scales are mostly the national level or the provincial level. However, due to historical and market economic development, there are significant differences in various factors such as economy, industrial structure, technological level, energy consumption and environment in various regions of China. In order to further carry out carbon emission research, this paper introduces gender factors and gender factors and GDP interactions at the national and regional levels, and then deeply study the effects of carbon emissions. (2) In the existing literature, there are few choices for spatial weight matrix, and most of the literature adopts the method of subjective choice determination, which cannot reflect the true relationship optimally. Sometimes it may be caused by improper selection of weight matrix. The model results in a bias. There are also subjective choices for the selection of spatial econometric models. The model SDM with SAR and SEM structures is generally selected as the analytical model, so that although the effects of different structures of different variables on carbon emissions can be comprehensively analyzed, SDM does not include SDEM model structure, if the real data structure conforms to SDEM or other non-SDM data structures, the use of SDM structures may over-interpret certain variables. (3) With the emphasis on dynamic issues by scholars, there are also selection problems in the data form of spatial measurement models. Which data types in static panel and dynamic panel data types are more able to reflect the meaning of variables? There are few references about the selection of this type of data.

Based on the above research, this paper discusses the impact mechanism of economics, gender factors and gender-based synergies on carbon emissions from the national level and regional levels, and sets up two data types, four alternative spatial measurement models and 12 kinds of optional spatial weight matrix, a total of 96 model structures, using Bayesian posterior probabilities and data-driven methods to select the optimal spatial weight matrix and econometric model, reducing the possibility of inappropriate selection of model structure due to subjective factors, improve the accuracy of the analysis results. The choice of control variables not only considers the gender factors in the population structure, but also selects factors such as aging factors, economic factors, industrial structure, and household consumption, fixed asset factors. It adds the interaction between economic and gender factors in a spatial perspective, from different levels. A more in-depth analysis of the impact of economics, gender factors and gender-based synergies effect on carbon emissions.

## 2. Research Method and Data

### 2.1. Construction of Spatial Econometric Model Structure

#### 2.1.1. Spatial Weight Matrix

The setting of spatial weight matrix is the most important in spatial econometrics. It mainly reflects the neighboring relationship between spatial units, which has a great influence on the estimation results of the model. At the same time, there are difficult attributes of selecting diversity. In order to be more objectively reflecting the relationship between spatial units, a total of 12 widely used spatial weight matrices are selected as candidate matrices, namely: (i) k-nearest neighbor matrix, knn = n (n = 1, …, 8), indicating that the n regions closest to the geographical location are neighboring relationships; (ii) the n-th order neighboring matrix (n = 1, 2) indicates that the geographic location is adjacent to the first order, and the first-order neighboring region is adjacent to the second-order neighboring relationship. The proximity relationship is divided into Queen Proximity and Rook Proximity. This matrix is often used in spatial measurement models and is easy to understand and explain. Details are shown in Table 1.

Here knn = n is the nearest neighbor spatial weight matrix, and the distance between each region and the central region is calculated, and then the distance values are sorted from small to large, and the first n values are selected, and the corresponding region indicates adjacent to the central region. Q1 and Q2 are Queen’s adjacent spatial weight matrix, and are defined adjacent to or adjacent to the edge of the central region, where Q1 represents first-order neighbors, that is, edges and corners are adjacent, and Q2 represents 2nd-order neighbors, that is, adjacent to the sides and corners of the adjacent area. R1 and R2 are Rook neighboring spatial weight matrices, and the definition is only adjacent to the edge of the central region, where R1 represents a first-order neighbor, that is, the edges are adjacent, and Q2 represents a second-order neighbor, that is, the edge with the adjacent region. The weight matrix of this paper is the location data of 30 provinces except Hong Kong, Macao, Taiwan and Tibet. Because Hainan is not close to Guangdong and Guangxi, the Hainan area is independent according to the weight matrix set by Queen and Rook. There is no adjacent area, so the geographical location of Hainan is adjusted to be adjacent to Guangdong and Guangxi, and the influential weights are adjusted.

#### 2.1.2. Spatial Econometric Model

This paper focuses on the mechanism of the impact of GDP based on GEN synergies on carbon emissions. According to Zhang et al. [38] and Friedrich [39], the basic model containing interaction terms is constructed first, as shown in Equation (1):

(1) $$CO2_{it}=\alpha +\beta_{1}ECO_{it}+\beta_{2}GEN_{it}+\beta_{3}ECO_{it}*GEN_{it}+\sum_{i=3}^{n}\beta_{i+1}x_{it}+\mu_{i}+\upsilon_{t}+\varepsilon_{it}$$

where $CO2_{it}$ is the dependent variable and represents carbon emissions in this paper; $ECO_{1t}$ is the economic factors; $GEN_{it}$ is the gender factor; $\sum_{i=3}^{n}x_{it}$ is the other control variable; $ECO_{it}\times GEN_{it}$ is the interaction term of the multiplication of $ECO_{it}$ and $GEN_{it}$, indicating the independent variable the interaction of variables; $\mu_{i}$ and $\upsilon_{t}$ respectively represent regional fixed effects and time fixed effects; $\varepsilon_{it}$ represents residual terms.

In order to accurately analyze the interaction effect of the independent variable on the dependent variable, the first-order partial derivative is obtained for the two sides of Equation (1), as shown in Equation (2):

(2) $$\frac{\partial CO2_{it}}{\partial ECO_{it}}=\beta_{1}+\beta_{3}GEN_{it}$$

The marginal effect of the independent variable $ECO_{it}$ on the dependent variable $CO2_{it}$ depends on the independent variable $GEN_{it}$. If $\beta_{3}>0$, the marginal effect of $ECO_{it}$ on $CO2_{it}$ increases with increasing $GEN_{it}$, which is the synergy effect. Conversely, if $\beta_{3}<0$, the marginal effect of $ECO_{it}$ on $CO2_{it}$ decreases as $GEN_{it}$ increases.

To find the first derivative of $GEN_{it}$ on both sides of (2), we can get the mediating effect of $GEN_{it}$ as a mediator on $ECO_{it}$ affecting $CO2_{it}$. The result is shown in Equation (3):

(3) $$\frac{\partial^{2}CO2_{it}}{\partial ECO_{it}\partial GEN_{it}}=\frac{\partial (\frac{\partial CO2_{it}}{\partial ECO_{it}})}{\partial GEN_{it}}=\beta_{3}$$

Therefore, the regression coefficient $\beta_{3}$ before the interaction term is also called “Interaction Effect”, or “Moderating Effect”, that is, the effect of $ECO_{it}$ on $CO2_{it}$ is affected by $GEN_{it}$. When $GEN_{it}$ is unchanged, the influence intensity of $ECO_{it}$ on $CO2_{it}$ is $\beta_{1}$ + $\beta_{3}$.

The spatial econometric model not only considers the relationship between each variable and the dependent variable, but also adds spatial features to more comprehensively analyze the intensity of each variable’s influence on the dependent variable. The paper selects the commonly used static and dynamic spatial panel model as alternative data type models. The static spatial panel model is based on the general form of the spatial linear model in spatial econometric analysis given by Anselin [40]. By adding the subscript t to the cross-section model [41], the observation value in the cross-section spatial measurement model can be obtained. The general nested spatial model of the cross section is expanded into a space-time model of panel data with observations and across periods, where *t* is from 1 to ${}_{T}$, it applies to all variables and error terms of the model, and which is extended to a model with a specific spatial effect or time effect, as shown in Equation (4):

(4) $$\begin{array}{l} Y_{it}=\rho WY_{it}+\alpha l_{i}+X_{it}\beta +WX_{it}\theta +\mu_{i}+v_{t}+u_{it}\\ u_{it}=\lambda Wu_{it}+\varepsilon_{it} \end{array}$$

where $Y_{it}$ represents the N×T dimension variable matrix, consisting of variable observations of each spatial unit i(i=1, …, N) in the sample at time t(t=1, …, T); $X_{it}$ represents the N×K dimensional independent variable matrix, K represents the number of independent variables, and W refers to the N×N dimensional space Weight matrix, α is a constant term parameter; $\mu_{i}=(\mu_{1},\ldots ,\mu_{N}{)}^{T}$ is a spatial-specific effect; $v_{t}={\left(v_{1},\ldots ,v_{T}\right)}^{T}$ is a time-specific effect; $\varepsilon_{it}={\left(\varepsilon_{1t},\ldots ,\varepsilon_{Nt}\right)}^{T}$ is an interference-dependent vector obeying the *iid* distribution.

With the emphasis on dynamics by scholars, a group of scholars such as Baltagi, Elhorst, and Li have made in-depth research on the estimation method of dynamic space panel models, which makes it possible to solve dynamic models on spatial problems. The general form of dynamic panel models is shown in Equation (5):

(5) $$\begin{array}{l} Y_{it}=\tau Y_{i,t-1}+\delta WY_{it}+\eta WY_{i,t-1}+X_{it}\beta_{1}+WX_{it}\beta_{2}+X_{i,t-1}\beta_{3}+WX_{i,t-1}\beta_{4}{+Z}_{it}\pi +\mu_{i}+v_{t}+\varepsilon_{it}\\ \varepsilon_{it}=\rho \varepsilon_{i,t-1}+\lambda W\varepsilon_{it}+\gamma W\varepsilon_{i,t-1}+\xi_{it} \end{array}$$

where: $Y_{t}$ represents the N×T dimensional dependent variable matrix; $X_{t}$ represents the N×K dimensional independent variable matrix, $Z_{t}$ is the N×L dimensional exogenous explanatory variable matrix; W refers to the N×N-dimensional spatial weight matrix, $\mu_{i}=(\mu_{1},\ldots ,\mu_{N}{)}^{T}$ is a spatial-specific effect; $v_{t}={\left(v_{1},\ldots ,v_{T}\right)}^{T}$ is a time-specific effect; $\varepsilon_{it}={\left(\varepsilon_{1t},\ldots ,\varepsilon_{Nt}\right)}^{T}$ is an interference-dependent vector, $\xi_{it}={\left(\xi_{1t},\ldots ,\xi_{Nt}\right)}^{T}$ is an interference-dependent vector obeying the *iid* distribution.

When selecting the model structure of static spatial panel model and dynamic spatial panel model, Spatial Autoregression model (SAR), Spatial Durbin model (SDM), Spatial Error model (SEM) and Spatial Durbin error model (SDEM) are selected as the alternative models under each type of data. Combining the static panel general model and the dynamic panel general model of equations (4) and (5), refer to the definition of each model in the existing literature, and the parameters of each model selected in this paper. The settings are shown in Table 2.

Spatial econometrics has strict requirements on data. Since the main part of the spatial metrological model is the spatial weight matrix, which is also the key and difficult part of the spatial metrological model, the use of the spatial metrological model must ensure the spatial correlation of the data, that is, the variables that measure the spatial correlation should represent the significant correlation of the space. Take the value of Moran’s I as an example. The value of Moran’s I must be greater than or less than 0 and significant

#### 2.1.3. Structure Selection of Bayesian Posterior Probability Model

Because the model structure is complex and there are many alternative model structures, how to choose the optimal model structure to study the relationship between variables becomes the difficulty and focus of the model setting. From the data-driven perspective, the spatial evolving model selection method based on Bayesian posterior probability [42,43], objectively selects the optimal model structure and reduces the error caused by subjective model selection. The paper refers to the existing literature [44,45], combining the alternative spatial weight matrix, the selected data type and the alternative spatial measurement model set above, calculating the Bayesian posterior probability of each model structure using MATLAB code written by Elhorst [44,45], and the model structure with the largest posterior probability is selected as the optimal model structure.

### 2.2. Data

Since there is no official statistics on carbon emissions in various provinces, the paper selects the carbon emissions measured by energy data as the target variable. The 2007 Intergovernmental Panel on Climate Change (IPCC) Fourth Assessment Report [46] pointed out that fossil fuel combustion is the main source of greenhouse gases (carbon emissions from fossil fuel combustion in 2004 accounted for 95.3% of the world’s total emissions), so this paper mainly selects fossil energy data. By referring to existing literature and documents, the paper finally selected eight types of energy consumption categories as the basis for the estimation of carbon emissions, namely coal, gasoline, diesel, natural gas, kerosene, fuel oil, crude oil and coke. When accounting for inter-provincial carbon emissions, the total energy consumption is converted into standard coal and multiplied by their respective carbon emission factors, and then the carbon emissions of various energy sources are aggregated. The estimation formula is shown in Equation (6):

(6) $$C_{it}=\sum \left(A_{ijt}\cdot E_{j}\cdot \eta_{j}\right)$$

where $C_{it}$ is the total carbon emissions in the t-th year of the i-th region, $A_{ijt}$ is the j-th energy consumption in the t-th year of the i-th region; $E_{j}$ is the conversion factor when the j-th energy is converted into standard coal; $\eta_{j}$ is the carbon emission factor of the j-th energy. The carbon emission factors of various energy sources are shown in Table 3.

Based on the availability of data, we can calculate the carbon emissions data of China’s 30 provinces except Hong Kong, Macao, Taiwan and Tibet in 2005–2016 based on the availability of data.

The selection of control group variables refers to the relevant research results of domestic and foreign scholars on the analysis of carbon emission and carbon emission influencing factors, not only the gender factors in the demographic factors, but also the secondary industry factors, economic factors, consumption factors and fixed asset factors. The definition of indicators and the description of variables are shown in Table 4. This article uses annual panel data for 2005–2016 in 30 provinces of China except Hong Kong, Macao and Taiwan and Tibet. The data are all from the Statistical Database of the China Statistics Bureau, *China Statistical Yearbook* and China Economic Net Statistics Database. In order to eliminate heteroscedasticity and other problems, this paper conducted logarithmic processing of all data, and found that the data after logarithmic processing were stable through testing.

## 3. Results

### 3.1. Impact of Variables at the National Level on Carbon Emissions

#### 3.1.1. Model Structure Selection Result

According to the selection method of spatial econometric model structure based on Bayesian posterior probability proposed by Lesage, 12 kinds of spatial weight matrices, four kinds of spatial econometric models and two kinds of data in this paper are calculated, and a total of 96 kinds of model structures are composed. The posterior probability of each model structure is shown in Table 5.

As shown in Table 5, the posterior probability of the spatial Durbin model (SDM) in the static panel model is 0.919, which is the largest among the four spatial metrological models and two data types. Therefore, the spatial Durbin model (SDM) in the static panel model is selected as the optimal spatial metrological model. According to the selected static panel space Durbin model (SDM), by comparing the values of 12 weight matrices, it is found that the posterior probability of knn = 8 is the largest among the 12 weight matrices, which is 0.919. Therefore, knn = 8 is selected as the optimal spatial weight matrix. Therefore, according to the selection results of model, data and spatial weight matrix, the GEN-based synergy effect is used to study the carbon emission research of ECO. The optimal model structure is a static panel spatial Durbin model based on the spatial weight matrix of knn = 8.

According to the selection result of the model structure, the model structure of the static panel SDM with double fixed effect is finally selected as the optimal model for exploring the problem (Equation (7)):

(7) $$Y_{t}=\rho WY_{t}+\alpha l_{N}+X_{t}\beta +WX_{t}\theta +\mu +v+\varepsilon_{t}$$

#### 3.1.2. Global Spatial Autocorrelation Test

**Figure 1:**
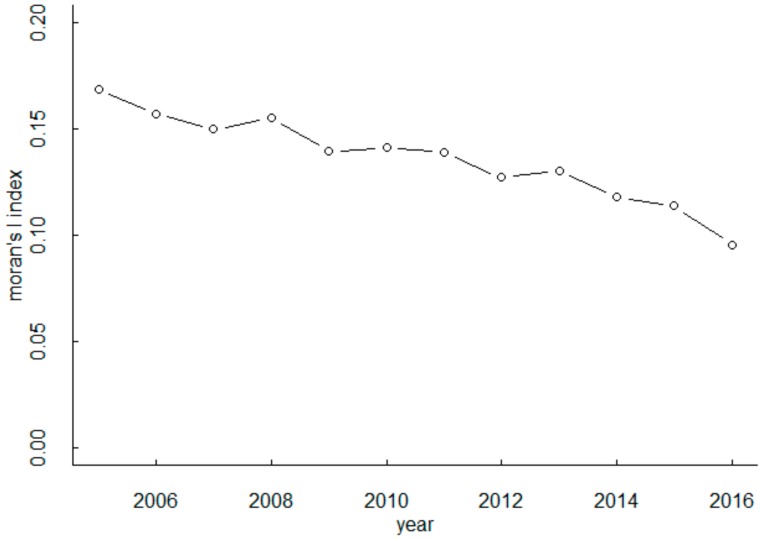
Trend of Moran’s I index.

The global spatial autocorrelation index is an indicator for judging whether it has spatial effects, it is also the basis for whether or not to add spatial features in the model, the Moran’s I index is generally chosen as the result reference. In the general research sequence, it is necessary to calculate the global spatial autocorrelation index of the data first, and then determine whether the data has spatial features and whether spatial features are added to the model. However, due to the order of the model structure selection in this paper, this paper first assumes that the data has spatial features, and then calculates the Moran’s I index of the data according to the selected optimal spatial weight matrix to prove whether the assumption is true. According to the selected optimal spatial weight matrix (knn = 8), the global spatial correlation of the carbon emission data of each region in the sample is tested by ArcGIS. The test results and the Moran’s I index change are shown in Table 6 and Figure 1.

The Moran’s I index indicates the extent of spatial agglomeration. From the test results in Table 6, it is known that the P value of each year is less than 0.01, indicating that the Moran’s I index passed the significance test. It can be seen from Figure 1 that the Moran’s I index is greater than 0, indicating that the carbon emissions in the studied range exhibit significant spatial agglomeration characteristics, and the spatial dependence characteristics gradually decrease with time.

#### 3.1.3. Model Estimation Result

According to the Bayesian posterior probability model selection method, the selected optimal model structure is determined as a static spatial Durbin panel model (SSDM) with double fixed effect based on the spatial weight matrix knn = 8. To further analyze the impact of ECO on carbon emissions and the synergy based on GEN, we first explore the effects of ECO on carbon emissions at the national level (Table 7).

Carbon emissions present a significant spatial spillover effect. As shown in Table 7, the spatial econometric optimal model structure selection method of Bayesian posterior probability can effectively select the optimal model structure. The selected SSDM model and the spatial weight matrix of knn = 8 can better fit the data. The goodness is 0.661, and the log-likelihood ratio is −318,869.650. By observing the spatial lag term of carbon emissions, the coefficient of W*CO2 is 0.740, which is significant at the 1% significance level. For every 1 unit of carbon emissions in the central region, the carbon emissions in the surrounding areas will increase by 0.740 units. Carbon emissions present a significant spillover effect in the spatial region.

The increase of ECO and GEN has contributed to the increase in carbon emissions in the region and has a significant negative impact on carbon emissions in surrounding areas. The coefficient of ECO is 0.825, and the coefficient of W*ECO is −1.945, both of which are significant at the significance level of 1%. For every 1 unit increase in ECO in the central region, the carbon emissions in the region will increase by 0.825 units, but the carbon emissions in the surrounding areas will decrease by 1.945 units. ECO has significantly contributed to the increase in carbon emissions in the region and has curbed carbon emissions in the surrounding areas. By analyzing the fixed effect of ECO on carbon emissions, it is found that the direct effect coefficient of ECO on carbon emissions is 0.609, the indirect impact effect is −4.770, and the total impact effect is −4.362, and both are significant at least at the significance level of 5%. The direct effect of ECO on carbon emissions is positive, but the indirect impact and total impact effect are negative, and the coefficient is larger than the direct impact effect, indicating that the indirect negative impact intensity of ECO on carbon emissions is significantly greater than the direct impact intensity, and significant positive correlation between ECO and carbon emissions is not entirely established. With the enhancement of regional cooperation and environmental regulation, the complementary and cooperative trend of inter-regional product production has been significantly enhanced, the supply of products in the region has eased the production of similar products in the surrounding areas, and the surrounding areas have reduced carbon emissions from production. The coefficient of GEN is 4.972, the coefficient of W*GEN is −4.111, and both are significant at least 5% of the significance level, for each unit of GEN increase, the carbon emission of the region will increase by 4.972 units, and carbon emissions in the surrounding areas will be reduced by 4.111 units, and the direct and total benefits of GEN are significantly positive. The GEN mainly represents the proportion of male and female population, while for China’s labor data, male labor is greater than female labor, especially in the secondary industry. The increase in the proportion of men has contributed to the development of economy in the region, and the rapid economic development has led to an increase in carbon emissions. The regions with high economic level have strong attraction to talents, which leads to the male labor force in the surrounding areas flowing to the central areas with high economic level, the number of male laborers in the surrounding areas is gradually decreasing, and the economic development is slowing down, resulting in a significant decline in carbon emissions trend.

The intensity of ECO’s impact on carbon emissions increases with the increase of GEN in the region, but decreases with the increase of GEN in the surrounding areas. The coefficient of ECO and GEN interaction ECO*GEN is 1.234, and the coefficient of W* ECO*GEN is −2.868, both of which are significant at the significance level of 1%. ECO and GEN have significant synergistic effects on carbon emissions, the intensity of ECO impact on carbon emissions increases with the increase of GEN. For every unit of GEN increase, the impact of ECO on carbon emissions will increase 2.059 units. The ECO and GEN factors in the surrounding areas have a significant inhibitory effect on the carbon emissions of the region. The intensity of the impact of ECO in the surrounding areas on the carbon emissions in the region has weakened with the increase of GEN in the surrounding areas. For each additional unit of GEN in the surrounding area, the impact of ECO in the surrounding area on the carbon emissions of the region will be reduced by 4.813. The GEN has a significant positive impact on the intensity of ECO impact on carbon emissions in the region, but significantly inhibits the intensity of ECO in the surrounding region. The male labor force has played a significant role in the growth of ECO, and most of the economic construction in which the male labor force participates is dominated by high carbon emission industries.

The economic development level of all provinces in China has shown an overall upward trend, at the same time, competition between them has become increasingly fierce. GEN play an important role in promoting labor competition, especially in neighboring provinces with homogeneous development. With the continuous green transformation and upgrading of industries, the ECO and GEN of neighboring provinces with gradient differences form positive externalities, and the improvement of the economic development level in this region brings about the decline of carbon emission intensity in the surrounding areas. Due to the openness of the region, different provinces have reduced the negative impact of pollutants and wastes on the surrounding provinces while carrying out pollution control, ecological construction, energy conservation and emission reduction to improve their ecological environment and civilization. In particular, in recent years, inter-provincial collaborative management of environmental issues between provinces has gradually increased, and the intensity of carbon emissions in surrounding areas has gradually declined.

### 3.2. The Impact of Various Variables on Carbon Emissions at the Regional Level

Due to the vast territory of China, the uncoordinated development of the Eastern, Central Region, Western and Northeast regions, and significant differences in economy, culture, science and technology, how will the ECO, GEN and “Synergies” based on GEN be affected by the background of such differences carbon emission? In order to more clearly analyze the impact of GEN on carbon emissions in different environments, this paper has divided the regions of China. This part uses the above methods to study different regions of China. For the division of each region, refer to Chen [48] for the inter-provincial classification of China. The 30 provinces except Hong Kong, Macao and Taiwan and Tibet of China are divided into Eastern, Central Region, Western and Northeastern regions. The detailed division is shown in Table 8.

According to the regional classification criteria of 30 provinces in China, the optimal model structure determined by the optimal structure selection method above is used to analyze the impact of ECO on carbon emissions in four regions and the impact of “synergy” based on GEN mechanism, the results are shown in Table 9.

Carbon emissions in the Eastern and Western regions have significant spillover effects, and carbon emissions in the Central Region and Northeastern region have a significant agglomeration effect. By comparing and analyzing the effects of regional variables on carbon emissions in Table 9, it is found that the fitting effects of each model are significant, and the optimality of model selection based on Bayesian posterior probability model selection method is verified again. From the coefficient of W*CO2 in Table 9, the coefficients in the Eastern and Western regions are significantly positive, and the coefficients in the Central Region and Northeastern region are significantly negative. The increase in carbon emissions in the Central Region has significantly increased carbon emissions in the surrounding areas in the Eastern and Western regions, but significantly suppressed carbon emissions in the surrounding areas in the Central Region and Northeastern region.

ECO and GEN have significantly increased carbon emissions in the region and suppressed carbon emissions in surrounding areas. As ECO and GEN increase, the impact on carbon emissions in the Eastern and Northeastern regions is significantly positive, and the intensity of GEN impacts on carbon emissions is significantly greater than the impact of ECO on carbon emissions. The impact of the spatial lag term of ECO on carbon emissions has a significant negative impact in the Eastern, Central Region and Northeastern regions. The effects of the spatial lag term of GEN on carbon emissions have a significant negative impact in the Eastern, Central Region, Western, and Northeastern regions. The increase in ECO and GEN has significantly increased carbon emissions in the region in the Eastern and Northeastern regions, but significantly inhibited carbon emissions in the surrounding regions.

The impact of ECO and GEN on carbon emissions presents significant synergies in the Eastern and Northeastern regions, with significant non-synergy effects in both the ECO and GEN and their lags in the Western region. The intensity of ECO impact on carbon emissions is increasing in the Eastern and Northeastern regions with increasing GEN. The greater the value of GEN, the stronger the impact of ECO on carbon emissions, and the significant synergistic effects of ECO and GEN on carbon emissions. However, the intensity of ECO impact on carbon emissions weakens in the Western region with increasing GEN, the greater the GEN, the weaker the impact of ECO on carbon emissions. The intensity of the impact of ECO in the surrounding areas on the carbon emissions in the region is weakened by the increase of GEN in the surrounding areas. The GEN reduces the impact of ECO in the surrounding areas on the carbon emissions of the region.

The labor force plays an important role in the economic development of the Eastern and Northeastern regions, the increase in labor force has promoted infrastructure construction and economic development in the Eastern and Northeastern regions. From the perspective of technological progress, with the industrial transformation and upgrading, the continuous optimization of energy structure and energy efficiency as the main driving force, the continuous reduction of carbon intensity is closely related to the development of GEN. The Eastern region has had a significant effect on technological progress in curbing carbon emissions, which has also significantly enhanced the synergy between ECO and GEN. However, the labor force has restrained the economic development of the Western region. With the massive loss of labor, it is not conducive to the economic development of the region. The influence of GEN on production is greater than the impact on consumption. However, the large outflow of labor force in the Western region directly leads to the lack of supply of labor in the production sector in the Western region, GEN significantly inhibit the impact of economic development on carbon emissions.

## 4. Discussion

This paper mainly studies the influence mechanism of ECO based on GEN synergy on carbon emissions, and uses Bayesian posterior probability to select the optimal model structure. The results showed that the influence intensity of ECO on carbon emissions increased with the increase of GEN in the National level and the Eastern and Northeastern regions, and the influence intensity of ECO on carbon emissions in the Central Region decreased with the increase of GEN in the surrounding regions. However, as the data in this paper is interprovincial data, the correlation difference between the municipal situation of the same province may be large, so this conclusion can only represent the situation of interprovincial region, and cannot completely depict the relationship between GEN, ECO and carbon emission of all cities. In addition, the variable of GEN in this paper is the ratio of the male population to the female population in the whole province. There is no gender factor data of the secondary industry or heavily polluting industry. Due to the large difference in the situation of population occupation, the influence intensity of the result is larger than the actual situation.

In the process of coping with climate change, gender quotas should be introduced to reasonably distribute the gender distribution in the industrial production process, and more women should participate in climate policy making, which can not only promote gender equality, but also enhance the impact of gender factors on economic development and environmental protection.

## 5. Conclusions

Based on the panel data of 30 provinces in China from 2005 to 2016, the Bayesian posterior probability optimal model structure selection method was used to select the optimal model structure for the data of different regions, and the influence of the ECO and synergistic effect of GEN on carbon emissions was studied from the National level and Eastern, Central Region, Western and Northeastern regions. The conclusions are as follows:

(1) The increase of ECO promotes the increase of carbon emission in this region, but it has a restraining effect on the carbon emission in the surrounding areas. At the National level, the impact effect of ECO on carbon emissions is significantly positive, and carbon emissions increase with the increase of ECO. However, the indirect negative impact intensity of ECO on carbon emissions is significantly greater than the direct impact intensity, indicating that carbon emissions do not fully show an upward trend with the increase of ECO.
(2) GEN have a significant positive effect on the region at the National level and the Eastern and Northeastern regions, but not significantly in other regions, and have a significant negative impact on carbon emissions in surrounding areas. The intensity of GEN influence on carbon emissions in the region at the National level, Eastern and Northeastern region is significantly positive, but the spatial lags of GEN in each region are significantly negative. With the increase of the intensity of GEN, the increase of carbon emissions in the National level, Eastern and Northeastern regions has been significantly promoted, but carbon emissions in surrounding areas have been inhibited in various regions.
(3) The influence intensity of ECO on carbon emission increases with the increase of GEN in the National level, Eastern and Northeastern regions, while the influence intensity of ECO of peripheral regions on carbon emission in Central Regions decreases with the increase of GEN in peripheral regions. ECO and GEN have a significant synergistic effect on carbon emissions in National level, Eastern and Northeastern regions. The influence intensity of ECO on carbon emissions increases with the increase of GEN, and GEN significantly enhance the relationship between ECO and carbon emissions. ECO and GEN have a significant non-synergistic effect in space. GEN plays an attractive role in economic development. The larger the number of GEN is, the higher the economic level will be, and the larger the carbon emission will be. Besides, it will attract the labor force in the surrounding areas, leading to the reduction of carbon emission in the surrounding areas.

According to the research results of this paper, we suggest that government departments should accelerate industrial upgrading and promote the construction of green ECO. High ECO growth is accompanied by high carbon emissions. Economic growth is at the expense of the environment and has not met the requirements of low-carbon development. Government departments should accelerate the green upgrading of industries, vigorously support the development of low-emission industries, and promote the construction and development of green ECO. In addition, relevant industries should improve the skill level of male labor force and reduce the influence intensity of gender factors between GDP and carbon emissions. Gender structure is the main influence factor of carbon emission, and at the national level, the increase of male population will bring high carbon emission to the surrounding areas, while women will reduce the high carbon emission to the surrounding areas. Improving the technical level of male labor force can fundamentally improve the quality and efficiency of labor force in economic construction, reduce employees in industries with high carbon emission, promote innovation and drive the development of green economy.

## Figures and Tables

**Table 1:** Alternative space weight matrix description.

Spatial Weight Matrix	Illustrate
knn = n	The nearest n nearest neighbors in the neighborhood of the region are adjacent.
(n = 1, 2, …, 8)	
Q1	The first order of an area adjacent to the edge or corner of a particular area serves as the neighborhood
Q2	The second order of an area adjacent to the edge or corner of a particular area serves as the neighborhood
R1	The first order of an area adjacent to the edge of a particular area serves as the neighborhood
R2	The second order of an area adjacent to the edge of a particular area serves as the neighborhood

**Table 2:** Spatial model structure parameter settings.

Static Panel Model		Dynamic Panel Model	
SAR	θ=λ=0	SAR	$\beta_{2}=\beta_{3}=\beta_{4}=\rho =\lambda =\gamma =0$
SDM	λ=0	SDM	ρ=λ=γ=0
SEM	ρ=θ=0	SEM	$\delta =\eta =\beta_{2}=\beta_{3}=\beta_{4}=0$
SDEN	ρ=0	SDEN	δ=η=0

**Table 3:** Carbon emission factors of various energy sources.

Types of Energy	Conversion Factor	Carbon Emission Coefficient
	(kg Standard Coal/kg)	(kg Carbon/kg Standard Coal)
coal	0.714	0.748
coke	0.971	0.113
crude oil	1.429	0.585
fuel oil	1.429	0.618
gasoline	1.471	0.553
kerosene	1.471	0.342
diesel	1.457	0.591
natural gas	13.300	0.448

Note: The standard statistic unit for natural gas is t standard coal/10,000 m^3^. The data is derived from *China Energy Statistical Yearbook* and other references [37,47].

**Table 4:** Meanings and explanations of indicators of control variables.

Variable Name	Variable Definition	Variable Declaration
*GEN*	Gender Factors	Ratio of male to female population
*ECO*	economic factors	The proportion of the gross regional product in the gross national product of the year
*IND*	Secondary industry factors	The secondary industry accounts for the proportion of the total industry in the region
*CONS*	Consumption factors	The proportion of total regional consumption in the total national consumption of the year
*ASS*	Fixed asset factors	The proportion of completed investment in fixed assets accounted for the total amount of total fixed assets investment in the country in that year

**Table 5:** Bayesian posterior probability optimal model selection result.

Weight Matrix	Static Panel Mode				Dynamic Panel Model			
	SAR	SDM	SEM	SDEM	SAR	SDM	SEM	SDEM
knn = 1	0	0	0	0	0	0	0	0
knn = 2	0	0.005	0	0.004	0	0	0	0
knn = 3	0	0	0	0	0	0	0	0
knn = 4	0	0	0	0	0	0	0	0
knn = 5	0	0	0	0	0	0	0	0
knn = 6	0	0	0	0	0	0	0	0
knn = 7	0	0	0	0	0	0	0	0
knn = 8	0	0.919	0	0.072	0	0	0	0
Q1	0	0	0	0	0	0.006	0	0.013
Q2	0	0	0	0	0	0.391	0	0.360
R1	0	0	0	0	0	0.006	0	0.013
R2	0	0	0	0	0	0.108	0	0.102
Column sum	0	0.924	0	0.076	0	0.512	0	0.489

**Table 6:** China’s 2005–2016 carbon emissions Moran’s I index.

Year	2005	2006	2007	2008	2009	2010	2011	2012	2013	2014	2015	2016
Moran’s *I*	0.168	0.157	0.150	0.155	0.139	0.141	0.139	0.127	0.130	0.118	0.114	0.095
*z*-Value	262.5	171.4	110.5	153.0	25.8	41.6	21.1	−74.3	−48.5	−148.7	−182.1	−332.4
*p*-Value	<0.01	<0.01	<0.01	<0.01	<0.01	<0.01	<0.01	<0.01	<0.01	<0.01	<0.01	<0.01

**Table 7:** Impact of ECO on carbon emissions: National level

Variable	Coefficient	Fixed Effect		
		Direct Effect	Indirect Effect	Total Effect
ECO	0.825 ***	0.609 **	−4.970 ***	−4.362 ***
	(2.625)	(1.967)	(−5.405)	(−4.646)
*GEN*	4.972 ***	4.962 ***	−1.663	3.299 *
	(2.949)	(2.983)	(−0.705)	(1.665)
ECO *GEN	1.234 ***	0.917 **	−7.278 ***	−6.361 ***
	(2.647)	(1.994)	(−5.569)	(−4.753)
W*ECO	−1.945 ***			
	(−5.199)			
W*GEN	−4.111 **			
	(−2.390)			
W* ECO *GEN	−2.868 ***			
	(−5.279)			
W*CO2	0.740 ***			
	(18.357)			
Model		SSDM		
Weight matrix		K8		
Period fixed effects		YES		
Space fixation effect		YES		
R^2^		0.661		
log-likelihood		−318,869.65		

Note: * indicates significant at the 10% level; ** indicates significant at the 5% level; *** indicates significant at the 1% level.

**Table 8:** China regional division standard.

	Eastern (10)	Central Region (6)	Western (11)	Northeast (3)
**Covering the provinces**	Beijing, Tianjin, Hebei, Shandong, Jiangsu, Shanghai, Zhejiang, Fujian, Guangdong and Hainan	Henan, Hubei, Hunan, Anhui, Jiangxi and Shanxi	Chongqing, Sichuan, Yunnan, Guizhou, Guangxi, Shaanxi, Gansu, Ningxia, Xinjiang, Qinghai and Inner Mongolia	Liaoning, Jilin and Heilongjiang

**Table 9:** Impact of ECO on carbon emissions: regional level.

Variable	China’s Four Major Regions			
	Eastern	Central Region	Western	Northeast
ECO	3.911 ***	0.516	−0.636	5.010 ***
	(4.558)	(0.746)	(−1.470)	(2.637)
*GEN*	21.998 ***	2.289	−3.008	24.449 **
	(4.726)	(0.491)	(−1.435)	(2.251)
ECO*GEN	5.765 ***	0.628	−1.607 **	6.754 **
	(4.640)	(0.610)	(−2.497)	(2.379)
W*ECO	−4.763 ***	−2.021 ***	−0.814	−3.826 *
	(-5.177)	(−2.679)	(−1.581)	(−1.781)
W*GEN	−19.687 ***	−17.664 ***	−10.580 ***	−27.419 ***
	(-3.971)	(−3.094)	(−4.177)	(−2.631)
W* ECO*GEN	−7.056 ***	−3.689 ***	−2.728 ***	−7.874 **
	(-5.105)	(−3.017)	(−3.395)	(−2.570)
W*CO2	0.569 ***	−0.315 *	0.374 ***	−0.236 **
	(7.724)	(−1.609)	(4.578)	(−2.442)
Model	SSDM	SSDM	SSDM	SSDM
Weight matrix	K4	K4	Q1	K1
Period fixed effects	YES	YES	YES	YES
Space fixation effect	YES	YES	YES	YES
R^2^	0.572	0.852	0.984	0.880
log-likelihood	−814,006.6	−49,315.969	−33,139.449	NaN

Note: * indicates significant at the 10% level; ** indicates significant at the 5% level; *** indicates significant at the 1% level; S indicates a static panel model in the model, and D indicates a dynamic panel model.
